# Comparative transcriptomic and physiological analyses unravel wheat source root adaptation to phosphorous deficiency

**DOI:** 10.1038/s41598-024-61767-z

**Published:** 2024-05-14

**Authors:** Daozhen Luo, Muhammad Usman, Fei Pang, Wenjie Zhang, Ying Qin, Qing Li, Yangrui Li, Yongxiu Xing, Dengfeng Dong

**Affiliations:** 1 Guangxi Key Laboratory of Agro-Environment and Agric-Products Safety, College of Agriculture, Guangxi University, Nanning, 530004 China; 2 Sugarcane Research Institute, Guangxi Academy of Agricultural Sciences, Nanning, 530007 China

**Keywords:** Plant molecular biology, Plant physiology, Plant stress responses, RNA sequencing

## Abstract

Phosphorus (P) is a crucial macronutrient for plant growth and development. Basic metabolic processes regulate growth; however, the molecular detail of these pathways under low phosphorous (LP) in wheat is still unclear. This study aims to elucidate the varied regulatory pathways responses to LP stress in wheat genotypes. Phenotypic, physiological, and transcriptome analyses were conducted on Fielder (P efficient) and Ardito (P inefficient) wheat genotypes after four days of normal phosphorous (NP) and LP stress. In response to LP, Fielder outperformed Ardito, displaying higher chlorophyll content-SPAD values (13%), plant height (45%), stem diameter (12%), shoot dry weight (42%), and root biomass (75%). Root structure analysis revealed that Fielder had greater total root length (50%), surface area (56%), volume (15%), and diameter (4%) than Ardito under LP. These findings highlight Fielder’s superior performance and adaptation to LP stress. Transcriptome analysis of wheat genotype roots identified 3029 differentially expressed genes (DEGs) in Fielder and 1430 in Ardito, highlighting LP-induced changes. Key DEGs include acid phosphatases (*PAPs*), phosphate transporters (*PHT1* and *PHO1*), *SPX*, and transcription factors (MYB, bHLH, and WRKY). KEGG enrichment analysis revealed key pathways like plant hormones signal transduction, biosynthesis of secondary metabolites, and carbohydrate biosynthesis metabolism. This study unveils crucial genes and the intricate regulatory process in wheat’s response to LP stress, offering genetic insights for enhancing plant P utilization efficiency.

## Introduction

Phosphorous (P) is a primary macronutrient central to plant growth and development^1^. Although soil frequently harbors abundant total phosphorus, its binding with aluminum and iron in acidic soils renders it unavailable to crops^2^. Utilizing phosphorus fertilizer is a key strategy to enhance crop yield. Despite implementing efficient phosphorus fertilization practices, plants can only absorb about 30% of the applied phosphorus, while the remainder is lost due to fixation and microbial processes. This situation has led to excessive fertilizer application, contributing to environmental problems such as water source eutrophication. Moreover, this issue is exacerbated by the limited availability of rock phosphorus reserves^3^. Hence, the study of phosphorus nutrition has become of utmost importance. The main focus involves developing cultivars with high phosphorus efficiency and comprehending the morpho-physiological and molecular mechanisms that enable their adaptation to low phosphorus conditions. This panorama is fundamental for sustainable agriculture.

Plants respond to phosphorus deficiency through morphological, physiological, molecular, and metabolic pathways, which enhance both phosphorus uptake from the soil and internal phosphorus utilization. The response to low phosphorus (LP) includes alterations in root system architecture (RSA) and symbiotic associations with arbuscular mycorrhizal fungi, collectively aiding in adaptation to LP stress^4^. Additionally, plants under LP exhibit increased synthesis and secretion of organic acids (OAs), purple acid phosphatases (PAPs), and phytases^3^. Phosphate deficiency activates phosphate sensing mechanisms and molecular responses, which involve the coordinated expression of phosphate starvation-response (PSR) genes and microRNAs (miRNAs) in plant tissues^5^. The PHT1 family of high-affinity phosphate transporters is at the core of phosphate absorption; 9 and 13 PHT1s have been found in *Arabidopsis* and rice. Functional investigations have been conducted utilizing mutant and transgenic plants of *PHT1* genes^6,7^. A universally conserved master regulator known as the transcription factor PHOSPHATE RESPONSE 1 (PHR1) coordinates the transcriptional activation of a significant portion of phosphate starvation-response (PSR) genes^8^. The nuclear proteins SYG/PHO81/XPR1 (SPX) serve as Pi-dependent competitive inhibitors of PHR1 activity in both *Arabidopsis* and rice^9,10^. Moreover, other Pi-responsive transcription factors like WRKY, MYB, and bHLH families have been identified in several plant species^11^. Furthermore, miR399 decreases the transcript level of *PHO2*, which encodes a ubiquitin-conjugating E2 enzyme involved in the ubiquitination and degradation of PHT proteins^12^.

Wheat (*Triticum aestivum* L., BBAADD, 2n = 6x = 42) is the most widely cultivated staple food crop^13^. In recent years, advancements have uncovered several molecular mechanisms governing Pi signaling and homeostasis in wheat. Genes encoding Al-activated malate and citrate transporters (such as TaALMT1 and TaMATE1B) play a crucial role in facilitating the efflux of organic acids (OAs), thereby improving the efficiency of Pi utilization in agricultural settings^14^. Phytases are phosphatases that can initiate the stepwise hydrolysis of phytate and thereby provide phosphate. The presence of phytase activity in the wheat can be attributed to three genes (*rTaPAPhy_a1*, *rTaPAPhy_b1*, and *rTaPhyIIa2*)^15^. In wheat, *TaPHT1;2*, *TaPht1;4*, *TaPHT1;9-4B*, and *TaPT2* (all members of the PHT1 family) facilitate Pi uptake under low-Pi conditions^16–19^, whereas *TaPHT2;1* (from the PHT2 family) and *TaPHO2-A1* are crucial for regulating Pi translocation^20,21^. Multiple transcription factors (TFs) play a role in wheat’s response to phosphate deficiency. For instance, TaPHR-A1, TaNFYA-B1, TaPHR3-A1-A, and TaMYB4-7D have been demonstrated to enhance the expression of several *TaPHT1* genes in transgenic wheat plants^19,22–24^. Furthermore, wheat miRNAs such as TaMIR1139 and TaMIR399 target genes across different families, playing pivotal roles in regulating plant tolerance to Pi starvation^20,25^. Metabolomic analysis presents a valuable tool in plant research for gaining deeper insights into plant responses to stress, particularly Pi starvation. This approach sheds light on the metabolic effects of Pi deficiency in wheat, revealing alterations in pathways related to carbohydrates, amino acids, and secondary metabolism^26,27^. Additionally, proteomic analysis revealed significant alterations in the abundance of proteins associated with nitrogen and phosphorus, small molecule, and carboxylic acid metabolic processes in response to Pi deficiency in wheat^28^. Plant hormones, such as auxin, cytokinin (CK), ethylene (ETH), abscisic acid (ABA), gibberellin (GA), jasmonic acid (JA), and salicylic acid (SA), undergo changes that play crucial roles in integrating Pi signaling and regulating root growth^29–31^. However, most studies have only investigated the effects of LP stress on one or two phytohormones in roots, with minimal available data on the comprehensive effects of LP stress on the phytohormone metabolome in wheat roots.

The plant’s response to low phosphorus (LP) stress is a multifaceted process regulated by the interplay of numerous genes. Despite this complexity, the transcriptional and metabolic mechanisms in wheat under LP stress remain relatively understudied. Advances in sequencing technologies, including metabolome and transcriptome analyses, offer promising avenues to accelerate the identification of molecular mechanisms governing plant responses to LP stress. This study delved into the physiological, transcriptomic, and targeted metabolomic responses of Fielder (P-efficient) and Ardito (P-inefficient) to elucidate key differentially expressed genes and metabolic pathways essential for LP response in wheat. These findings establish a fundamental groundwork and serve as a valuable resource for further exploration of gene regulatory mechanisms and a deeper understanding of the molecular processes involved in LP response and adaptation.

## Result

### Phenotypic changes and phosphorus efficiency under LP stress

**Figure 1:**
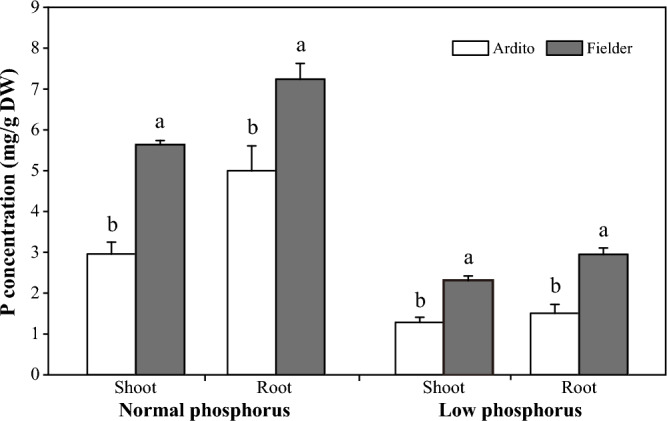
Total P concentrations were shown in the shoot and root under different levels of Pi-supply in two wheat genotypes. DW, dry weight. Values are means ± standard errors (n = 3). Different lowercase letters indicate significant differences (*p* < 0.05) within each index.

After undergoing low phosphorus (LP) treatment, two wheat genotypes experienced significant (*p* < 0.05) reductions in plant growth. Parameters such as SPAD, plant height, root dry weight, and shoot dry weight were significantly lower following LP treatment compared to normal phosphorus (NP) treatment in both genotypes (Table 1). Root structure analysis revealed decreased total root length, surface area, volume, and diameter in both genotypes under LP stress (Table 2). Notably, Fielder exhibited increased phenotypic indices under low-P stress compared to Ardito. Root images depicted elongated roots (greater root length) in Fielder compared to Ardito under LP (Figure S1). Overall, Fielder demonstrated superior performance to Ardito under LP stress, as evidenced by higher values and lower degradation rates of phenotypic indicators. Regarding root P contents, Fielder and Ardito experienced reductions of 59% and 70% under LP compared to NP, with Fielder showing a 95% increase in roots compared to Ardito. Additionally, shoots exhibited a 59% reduction in Fielder and a 56% reduction in Ardito under LP compared to NP, while shoot P was 79% higher in Fielder than Ardito under LP. Fielder accumulated more total P in both shoots and roots than Ardito, particularly in the roots (Fig. 1). These findings underscore Fielder's enhanced tolerance to Pi starvation and efficient Pi accumulation, designating it as the P-efficient line, while Ardito exhibited lower phosphorus tolerance.

**Table 1:** Effects of wheat on phenotypic changes under low phosphorus stress.

Pi-supply	Genotype	SPAD	Plant height (cm)	Stem diameter (mm)	Shoot dry weight (mg)	Root dry weight (mg)	Root to shoot ratio
NP (0.2 mM)	Fielder	37.87 ± 0.15a	39.12 ± 0.05a	1.50 ± 0.02a	45.87 ± 0.32a	10.87 ± 0.45a	0.23 ± 0.01ab
	Ardito	34.70 ± 0.26c	31.26 ± 0.32c	1.38 ± 0.02b	37.70 ± 2.75b	7.47 ± 0.15b	0.20 ± 0.01b
LP (2 µM)	Fielder	36.43 ± 0.31b	35.73 ± 0.24b	1.39 ± 0.01b	32.29 ± 2.17c	8.53 ± 0.55c	0.27 ± 0.03a
	Ardito	32.27 ± 0.32d	24.72 ± 0.50d	1.24 ± 0.02c	22.71 ± 0.79d	4.87 ± 0.60d	0.21 ± 0.02b

Values are means ± standard errors (n = 3). Different lowercase letters indicate significant differences (*p* < 0.05) within each index.

**Table 2:** Effects of wheat on root morphology under low phosphorus stress.

Pi-supply	Genotype	Total root length (cm)	Total root surface area (cm^2^)	Total root volume (cm^3^)	Root diameter (mm)
NP (0.2 mM)	Fielder	170.37 ± 10.75a	28.50 ± 2.81a	0.33 ± 0.06a	0.49 ± 0.03a
	Ardito	129.43 ± 5.56b	18.53 ± 1.31c	0.26 ± 0.01b	0.48 ± 0.02ab
LP (2 µM)	Fielder	161.80 ± 7.18a	24.07 ± 1.81b	0.23 ± 0.02bc	0.48 ± 0.01ab
	Ardito	107.57 ± 6.78c	15.80 ± 1.75c	0.20 ± 0.01c	0.46 ± 0.01b

Values are means ± standard errors (n = 3). Different lowercase letters indicate significant differences (p < 0.05) within each index.

### Wheat root transcriptome profiling in response to LP stress

**Figure 2:**
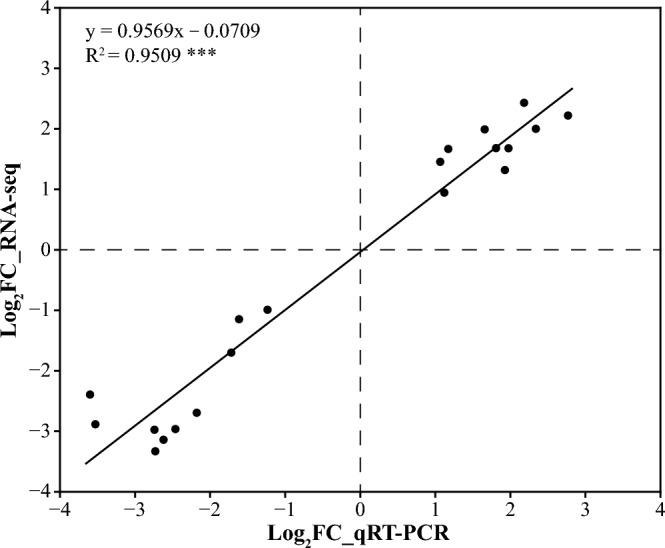
Correlation analysis of differentially expressed genes (DEGs) expression between transcriptome data and qRT-PCR results.

Transcriptome sequencing analysis was applied to investigate the molecular responses of wheat roots to Pi starvation. Twelve cDNA libraries were constructed, combining two genotypes, two treatments, and three replicates. These libraries yielded an average of 51.88 M clean reads (ranging from 44.29 M to 61.88 M). Additionally, a total of 113.40 G clean bases were generated, with respective lowest, highest, and average clean base values of 6.47 G, 8.85 G, and 7.56 G. Quality control data exhibited a Phred quality score Q30 ranging from 92 to 93.22%, coupled with an average GC content of 54.32%. Importantly, all samples demonstrated total mapping and uniquely mapping rates of 91.38% and 84.12%, respectively (average values), affirming the high quality and suitability of the sequencing data for subsequent gene expression analysis (Table S1). A comprehensive assembly yielded 118095 expressed genes (91852 known and 26243 new) and 192506 transcripts (113961 known and 78545 new). Subsequently, 91313 genes and 113446 transcripts were annotated by referencing KEGG, Swiss-Prot, Pfam, GO, COG, and NR databases. Principal component analysis (PCA) distinguished the 12 samples into two groups, Fielder and Ardito, effectively capturing differences between NP and LP treatments. PC1 and PC2 explained 18.34% and 39.70% variations, respectively (Fig. S2). The results ensured accurate replication within each group and highlighted distinct differences between genotypes and treatments. To validate RNA-Seq gene expression accuracy, qRT-PCR was conducted on 20 randomly selected DEGs (Table S2) using identical cDNA templates. The results exhibited a highly significant positive correlation with transcriptome data, affirming the reliability of the RNA-Seq results (Fig. 2).

### Identification of differentially expressed genes in response to LP stress

**Figure 3:**
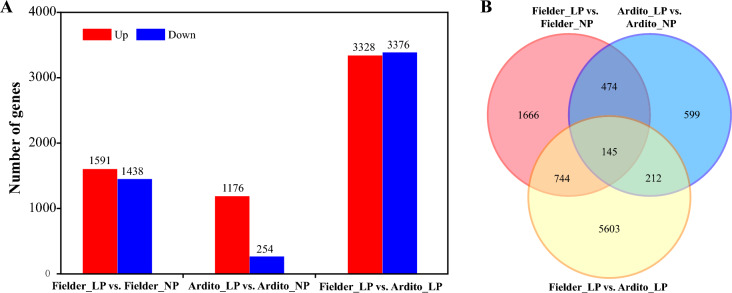
Differentially expressed genes profile for RNA-seq under LP stress in wheat. DEGs between low phosphorus (LP) and normal phosphorus (NP) of two constrasting varieties and DEGs between varieties under LP level (A) and Venn analysis to the DEGs (B).

Pairwise comparisons of genotype and treatment identified genes associated with Pi starvation. Fielder exhibited 3029 DEGs (1591 upregulated and 1438 downregulated), while Ardito had 1430 DEGs (1176 upregulated and 254 downregulated) compared to NP treatment. Surprisingly, Fielder had more DEGs than Ardito. P efficient genotype Fielder had 6704 DEGs (3328 upregulated and 3376 downregulated) compared to P inefficient genotype Ardito under LP stress (Fig. 3 and Table S3).

### GO annotations and KEGG enrichment analysis

**Figure 4:**
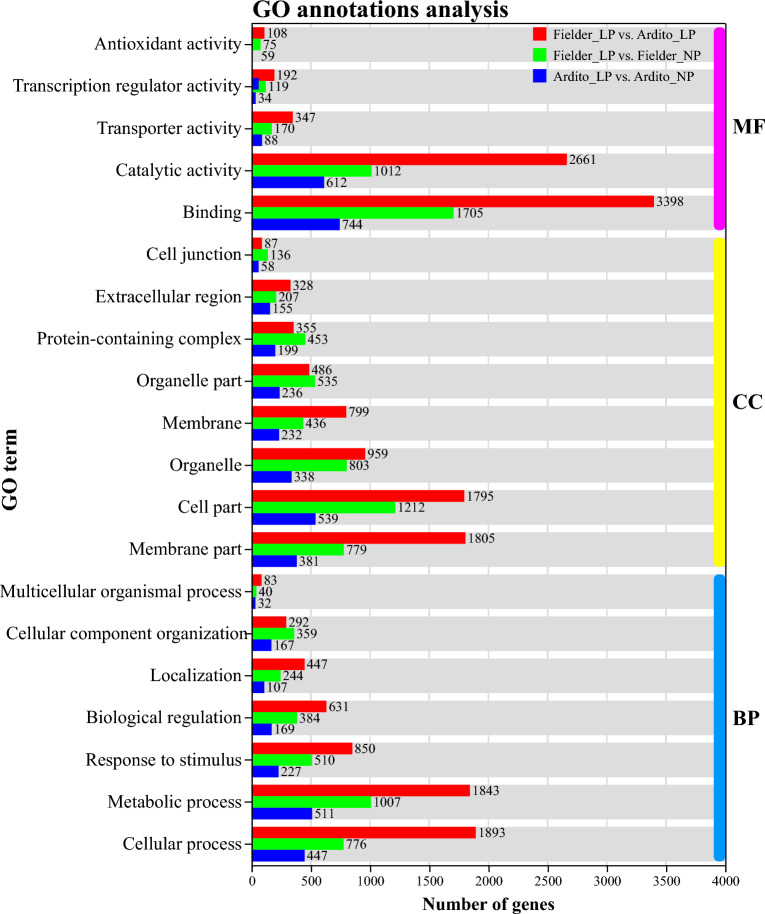
Top 20 GO annotations terms of DEGs under LP stress in wheat. *MF* molecular function, *CC* cellular component, *BP* biological process.

We performed GO annotation and KEGG pathway enrichment analyses of the DEGs identified in wheat under LP stress. In total, 3029 DEGs were grouped in 45 terms in Fielder, 1493 DEGs were grouped in 44 terms in Ardito, and 6704 DEGs were grouped in 47 terms in the comparison Fielder with Ardito were discovered in the GO database. Metabolic process (GO: 0008152), cellular process (GO: 0009987), and response to stimulus (GO: 0050896) were the most abundant GO terms annotated in the biological process (BP) in the three comparison groups (Table S4). Furthermore, membrane part (GO: 0044425), cell part (GO: 0044464), and organelle (GO: 0043226) were the most abundant GO terms in the cellular component (CC) (Table S4). Finally, binding (GO: 0005488) and catalytic activity (GO: 0003824) were the most enriched GO terms in the molecular function (MF) category (Fig. 4 and Table S4).

**Figure 5:**
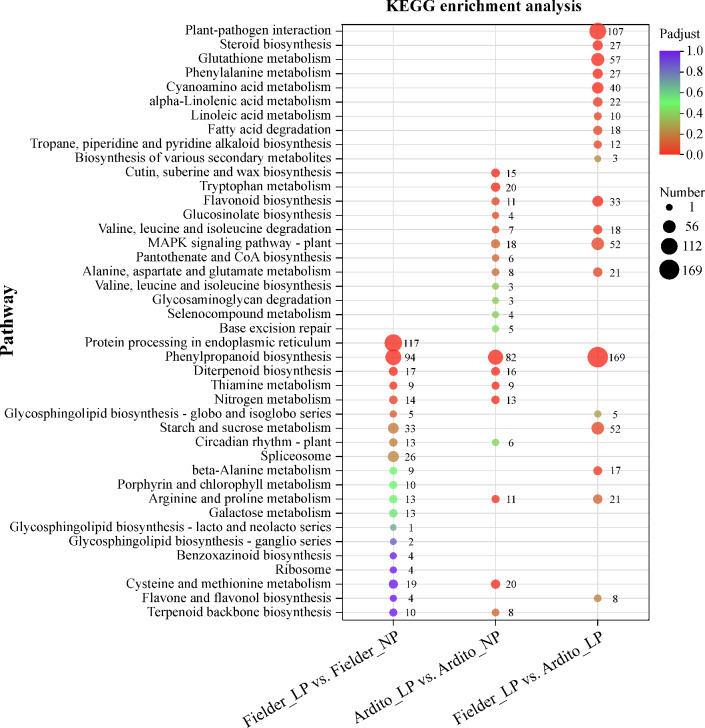
Top 20 enriched KEGG pathways of DEGs under LP stress in wheat. The bubble size indicates the number of DEGs involved in the pathways. *NP* normal phosphorus, *LP* low phosphorus.

The next step is to understand the functions of these significantly enriched DEGs. The KEGG pathway enrichment analysis showed that under LP stress conditions, the top 20 enriched KEGG pathways in Fielder, Ardito, and Fielder vs. Ardito are presented in Fig. 5. DEGs from three comparisons were enriched in two identical pathways, 94 genes in Fielder, 82 genes in Ardito, and 169 genes in Fielder vs. Ardito under LP stress were significantly enriched in phenylpropanoid biosynthesis (ko00940) pathway; 13 genes in Fielder, 11 genes in Ardito, and 21 genes in Fielder vs. Ardito were enriched in arginine and proline metabolism (ko00330) pathway. In addition, for Fielder, protein processing in the endoplasmic reticulum (ko04141, 117 genes), diterpenoid biosynthesis (ko00904, 17 genes), and nitrogen metabolism (ko00910, 14 genes) were the three main pathways. For Ardito, tryptophan metabolism (ko00380, 20 genes), Cysteine and methionine metabolism (ko00270, 20 genes), and diterpenoid biosynthesis (ko00904, 16 genes) were the three main pathways with many DEGs. For the comparison of Fielder with Ardito, plant-pathogen interaction (ko04626, 107 genes), glutathione metabolism (ko00480, 57 genes), MAPK signaling pathway-plant (ko04016, 52 genes), and starch and sucrose metabolism (ko00500, 52 genes) were the enriched pathways with the highest number of DEGs (Fig. 5). The differential expression of genes in response to LP stress-activated varied molecular mechanisms in various lines, as revealed by enriched KEGG pathways.

### DEGs related to pulper acid phosphatase and phosphate transporter

**Figure 6:**
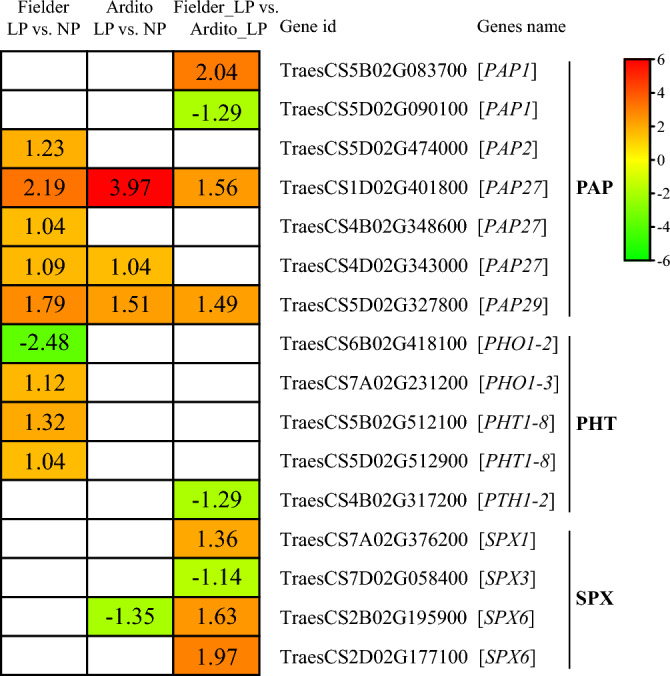
DEGs involved in purple acid phosphatase and phosphate transporter were identified in the RNA-seq analysis that showed changes in expression under LP stress in wheat. The numbers in the black rectangles show Log_2_(fold-changes) of the comparisons. The comparisons show that each gene in the picture is significantly differentially expressed. *PAP* purple acid phosphatase, *PHT, PHO* phosphate transporter, *SPX* SPX-domain-containing protein.

The transcriptome data revealed the identification of seven differentially expressed genes (DEGs) that encode pulper acid phosphatase (PAP) under LP stress (Fig. 6 and Table S5). Among these, five *PAP* genes, including one *PAP2*, three *PAP27*, and one *PAP29*, were significantly upregulated in Fielder; two *PAP7* and one *PAP29* genes were upregulated in Ardito under LP stress. One *PAP1*, one *PAP27*, and one *PAP29* genes were upregulated, and one *PAP1* gene was downregulated in Fielder compared with Ardito under LP stress. Four genes encoding phosphate transporter (PHT), including two *PHT1-8* and one *PHO1-3*, were upregulated, and one *PHO1-2* was downregulated in Fielder, but no response in Ardito. In addition, one *SPX1* and two *SPX6* genes were upregulated, and one *SPX3* gene was downregulated in Fielder compared with Ardito under LP stress (Fig. 6 and Table S5).

### DEGs related to plant hormones signal transduction

**Figure 7:**
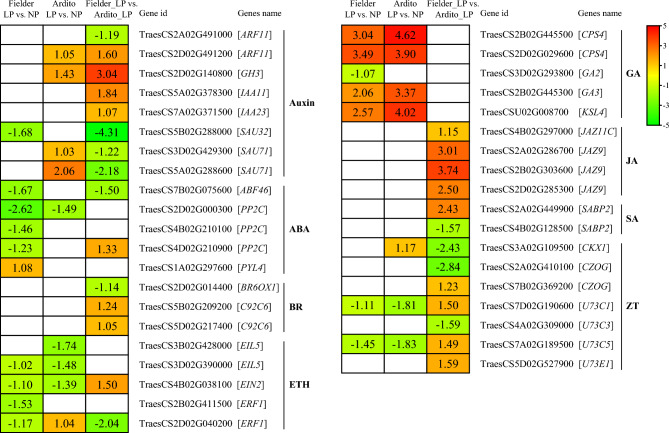
DEGs involved in plant hormone signal transduction in the RNA-seq analysis showed changes in expression during LP stress in wheat. *ARF* auxin response factor, *GH* indole-3-acetic acid-amido synthetase GH3, *SAU* auxin-responsive protein SAU, *ABF* abscisic acid factor, *PP2C* protein phosphatase 2C, *PYL* abscisic acid receptor PYL, *BR* brassinosteroid, *C92C* cytochrome P450, *EIL* ethylene insensitive 3-like protein, *ERF* ethylene-responsive transcription factor, *CPS* syn-copalyl diphosphate synthase, *GA* gibberellin, *KSL* terpene synthase, *JAZ* jasmonate ZIM-domain, *SABP* salicylic acid binding protein, *CZOG* *cis*-zeatin *O*-glucosyltransferase, *CKX* cytokinin dehydrogenase, *U37* UDP-glycosyltransferase.

The KEGG enrichment analysis showed that 52 DEGs enriched in the MAPK signaling pathway were identified under LP stress. Thirty-nine DEGs involved in auxin, abscisic acid (ABA), brassinosteroid (BR), ethylene (ETH), gibberellin (GA), jasmonic acid (JA), salicylic acid (SA), and zeatin (ZT) signaling pathways were identified to be associated with plant hormone signal transduction (Fig. 7 and Table S6). In the auxin signaling pathway, one *ARF*, one *GH3*, and two *IAA* genes were upregulated, but one *ARF* and three *SAUR* genes were downregulated in Fielder compared with Ardito under LP stress. One *ARF*, one *GH3*, and two *SAUR* genes were upregulated in Ardito, but one *SAUR* gene was downregulated in Fielder. In the abscisic acid signaling pathway, one *PYL* and one *PP2C* genes were upregulated in Fielder and Fielder vs. Ardito, respectively, while three *PP2C* and one *ABF* genes were downregulated. In the brassinosteroid signaling pathway, two *C92C* genes were downregulated, and one *BR* gene was downregulated in Fielder compared with Ardito. One *ERF1* and one *EIN2* genes were upregulated in the ethylene signaling pathway in Ardito and Fielder vs. Ardito. In contrast, two *EIN* and two *ERF1* genes in Fielder, three *EIN* genes in Ardito, and one *ERF1* gene in Fielder vs. Ardito were downregulated. In the gibberellin signaling pathway, two *CPS4*, one *GA3*, and one *KSL4* genes were upregulated in Fielder and Ardito, while one *GA2* gene was downregulated in Fielder. In the jasmonic acid signaling pathway, four *JAZ* genes were upregulated in Fielder compared with Ardito. In the salicylic acid signaling pathway, one *SABP2* gene was upregulated, and one *SABP2* gene was downregulated in Fielder compared with Ardito. In the zeatin signaling pathway, four *U73C* and one *CZOG* genes in Fielder compared with Ardito and one *CKX1* gene in Ardito were upregulated under LP stress. Two *U73C* genes were significantly downregulated in Fielder and Ardito, and one *CKX1*, one *CZOG*, and one *U73C3* genes in Fielder compared with Ardito were downregulated under LP stress (Fig. 7 and Table S6).

### DEGs related to secondary metabolic synthesis

**Figure 8:**
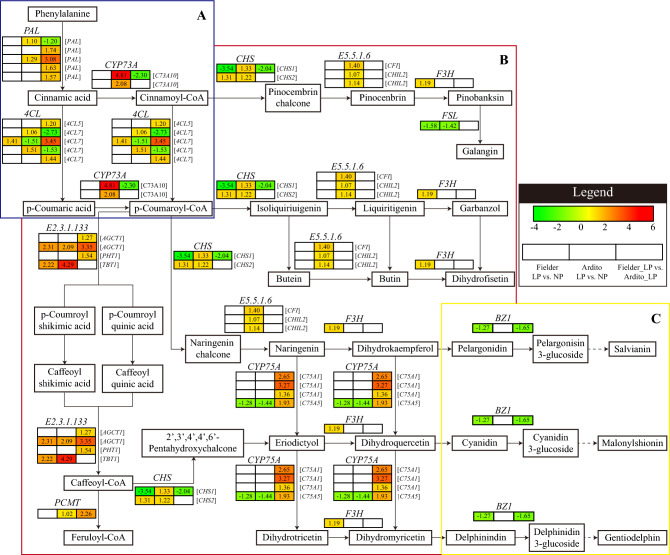
DEGs involved in secondary metabolic synthesis in the RNA-seq analysis showed changes in expression during LP stress in wheat. (**A**) The pathway of phenylpropanoid metabolism. (**B**) Flavonoid metabolism. (**C**) Anthocyanin metabolism. *PAL* phenylalanine ammonia-lyase, *CYP73A, CYP75A* cytochrome P450, *4CL* 4-coumarate-CoA ligase, *E2.3.1.133* Shikimate *o*-hydroxycinnamoyltransferase, *ACT* agmatine coumaroyl transferase, *PHT* putrescine hydroxycinnamoyl transferase, *TBT1* tryptamine benzoyl transferase, *PCMT* caffeoyl-CoA *O*-methyltransferase, *CHS* chalcone synthase, *E5.5.1.6* chalcone-flavanone isomerase, *F3H* naringenin,2-oxoglutarate 3-dioxygenase, *FLS* flavonol synthase/flavanone 3-hydroxylase, *BZ* anthocyanidin 3-*O*-glucosyltransferase.

Twenty-nine critical DEGs involved in secondary metabolic synthesis were significantly expressed in three comparisons, Fielder_LP vs. NP, Ardito_LP vs. NP, and Fielder vs. Ardito under LP stresses (Fig. 8 and Table S7). In the pathway of phenylpropanoid metabolism, four *PAL*, one *4CL5*, and two *4CL7* genes were upregulated, while one *PAL*, two *4CL7*, and one *C73A10* genes were downregulated in Fielder vs. Ardito under LP stress, respectively. One *4CL7* gene in Fielder, two *PAL*, and two *C73A10* genes in Ardito were upregulated under LP stress. Most structural genes implicated in the flavonoid metabolism pathways were significantly upregulated. For instance, four genes encoding shikimate o-hydroxycinnamoyltransferase (EC 2.3.1.133), four *CYP75A* genes, three genes encoding chalcone-flavanone isomerase (EC 5.5.16), two *CHS*, one *CAMT*, and one *F3H* genes were upregulated. Moreover, one *CHS1* gene was downregulated in Fielder and Fielder vs. Ardito. One *FLS* and one *C75A5* genes were downregulated in Fielder and Ardito. In the pathway of Anthocyanin metabolism, one *BZ1* gene was downregulated in Fielder and Fielder vs. Ardito under LP stress (Fig. 8 and Table S7).

### DEGs related to carbohydrate metabolism

**Figure 9:**
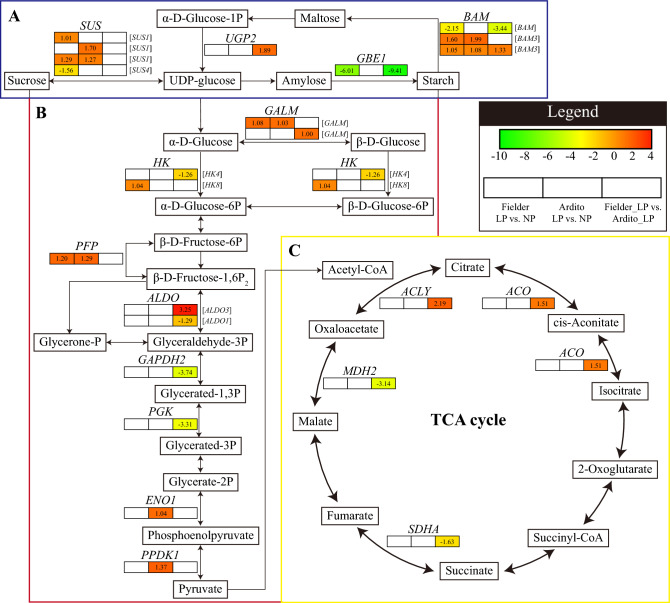
DEGs involved in carbohydrate metabolism in the RNA-seq analysis showed changes in expression during LP stress in wheat. (**A**) The pathway of starch and sucrose metabolism. (**B**) Glycolysis/gluconeogenesis. (**C**) Citrate cycle (TCA cycle). *SUS* sucrose synthase, *UGP* UTP-glucose-1-phosphate uridylyltransferase, *BAM* beta-amylase, *GBE* 1,4-alpha-glucan-branching enzyme, *GALM* galactose mutarotase, *HK* hexokinase, *PFP* pyrophosphate–fructose 6-phosphate, *ALDO* fructose-bisphosphate aldolase, *GAPDH* glyceraldehyde-3-phosphate dehydrogenase, *PGK* phosphoglycerate kinase, *ENO* enolase, *PPDK* pyruvate, phosphate dikinase, *SDHA* succinate dehydrogenase, *MDH* malate dehydrogenase, *ACLY* ATP-citrate synthase beta chain protein, *ACO* aconitate hydratase.

Twenty-eight crucial DEGs involved in carbohydrate metabolism were significantly expressed in three comparisons under LP stress (Fig. 9 and Table S8). In the starch and sucrose metabolism pathway, three *SUS* genes were upregulated in Fielder and Ardito, and one *SUS4* gene was downregulated in Fielder under LP stress. One *UGP2* gene was upregulated in Fielder vs. Ardito under LP stress. One *GBE1* gene was significantly downregulated in Fielder under LP stress. Two *BAM3* genes were upregulated, and one *BAM* gene was downregulated. In the glycolysis pathway, one *GALM* gene was upregulated in Fielder and Ardito and Fielder vs. Ardito under LP stress, respectively. One *HK4* gene in Fielder vs. Ardito was downregulated, and one *HK8* gene in Fielder was upregulated. One *PFP* gene was upregulated in Fielder and Ardito under LP stress. One *ALDO3* gene was upregulated, and one *ALDO1* gene was downregulated in Fielder vs. Ardito. One *GAPDH2* and one *PGK* genes were downregulated in Fielder vs. Ardito. One *ENO1* and one *PPDK1* genes were upregulated in Ardito. Genes involved in the citrate cycle (TCA cycle) pathway were significantly expressed in the comparison Fielder with Ardito, but no response in Fielder and Ardito comparison LP with NP, including the upregulation of one *ACLY* gene and one *ACO* gene and the downregulation of the *MDH2* gene and *SDHA* gene.

### DEGs related to transcription factors (TFs)

GO analysis results showed that numerous DEGs involved in transcription factors (TFs) were annotated in the GO database in the present study. A total of 426 TFs from 29 families were significantly expressed in three comparisons under LP stress. MYB, AP2, and bHLH were the top three leading TF families, containing 82, 50, and 44 differentially expressed genes (Table 3 and Table S9). Three TFs MYB (TraesCS7D02G295400), bHLH (TraesCS5A02G049600), and NAC (TraesCS2A02G101900) families were expressed in three comparisons which showed a downward trend. However, numerous TFs showed different trends in the two cultivars under LP stress. For instance, for the WRKY family, two genes were upregulated, and one gene was downregulated in Fielder; four genes were upregulated, and one gene was downregulated in Ardito; but in Fielder comparison with Ardito, nineteen genes were upregulated, and three genes were downregulated under LP stress. For the HSF family, twenty-three genes were downregulated, and one gene was upregulated in Fielder; six genes were upregulated, and two genes were downregulated in Fielder vs. Ardito, but no significantly responded in Ardito under LP stress. In addition, two genes from the SPB family, two from the RAV family, and one from the Nin-like family were exclusively downregulated in Fielder vs. Ardito under LP stress. One gene from NF-YA was exclusively upregulated, and one gene from ZF-HD was exclusively downregulated in Fielder under LP stress, respectively. One gene from E2F/DP was upregulated in Ardito under LP stress (Table 3 and Table S9). These transcription factors showed specialty in three comparisons.

**Table 3:** Differentially expressed transcriptional factors (TFs) under LP stress in wheat.

TFs family	Fielder_LP vs. Fielder_NP		Ardito_LP vs. Ardito_NP		Fielder_LP vs. Ardito_LP		Total
	Up	Down	Up	Down	Up	Down	
MYB	10	27	9	8	19	27	82
AP2	8	20	3	2	9	10	50
bHLH	7	10	4	5	16	13	44
WRKY	2	1	4	1	19	3	30
HSF	1	23	0	0	6	2	28
NAC	4	7	3	4	8	6	28
bZIP	3	3	1	2	9	12	26
HB	5	6	1	0	1	8	19
Dof	3	10	0	3	2	2	17
GRAS	1	0	0	0	6	9	16
DBB	5	8	0	5	1	1	15
B3	0	0	1	0	4	10	15
MIKC	0	3	0	0	2	4	8
FAR1	0	2	0	0	5	0	7
GATA	3	1	0	0	2	2	6
C3H	1	2	0	2	2	1	6
M_type	0	3	0	3	1	0	6
LBD	1	4	0	0	1	0	6
ARF	0	1	0	1	2	2	5
EIL	0	1	0	2	0	0	2
SBP	0	0	0	0	0	2	2
SRS	0	1	0	0	0	1	2
RAV	0	0	0	0	0	2	2
ZF-HD	0	1	0	0	0	0	1
NF-YA	1	0	0	0	0	0	1
E2F/DP	0	0	1	0	0	0	1
Nin-like	0	0	0	0	0	1	1
Total	55	134	27	38	115	118	426

### Phytohormone profiles in wheat roots

To confirm the transcriptomic findings, we performed targeted metabolomics analysis on 41 plant hormones in the roots, detecting 34 of them, including 7 AUXs (IAA, IAAla, IAAsp, IALeu, IAPhe, IATrp, and IPA), ABA, 6 GAs (GA1, GA3, GA4, GA7, GA8, and GA9), 2 GA synthesis inhibitors (UCZ and PP333), 3 JAs (JA, MeJA, and OPDA), SA, 10 CKs (mT, cZ, cZR, tZ, tZR, DZ, iP, iPR, 6-BA, and DPU), and 4 other plant growth regulators (MH, 1-NAD, 4-CPA, and 5-NG) (Table 4). The results were consistent with the transcriptome data. LP stress significantly increased the total auxin, total JA, and SA contents but decreased the total ABA and total CK contents in Fielder. Additionally, the total GA and SA increased, while the total IAA, CK, JA, and ABA content decreased in Ardito under LP stress. Furthermore, compared to Ardito, the total IAA, ABA, GA, JA, and SA content increased in Fielder under LP stress (Table 4). Regarding auxin, the IAA and IALeu content significantly increased, while the IAPhe content decreased in Fielder. The content of IAA has been increased by more than 2-fold in Fielder under LP stress. In contrast, the IAA, IAAsp, IAAleu, IAPhe, and IATrp content significantly decreased but increased the IAAla content in Ardito. As for GA, the GA1 and GA8 contents significantly increased, but the GA3 content decreased in Fielder. In Ardito, the GA7, GA8, and GA9 contents significantly increased, while the GA1, GA3, and GA4 content decreased. In terms of JA, the JA and OPDA content significantly increased, while MeJA decreased in Fielder. Conversely, the MeJA content decreased in Ardito, and the JA and OPDA content showed no significant influence. The total CK content accounts for about 80% of the total plant hormone content in the wheat root, with the highest being cZR. The CZ and DZ content increased, while the mT, CZR, and tZ content decreased in Fielder. In Ardito, the tZ content increased, while the cZ, cZR, tZR, and 6-BA content decreased. LP stress significantly decreased the MH and 4-CPA content in Fielder. Interestingly, 1-NAD was detected in Ardito under LP stress (Table 4).

**Table 4:** Concentrations (mean ± SE, n = 3) of 34 hormones detected under LP and NP treatment in two wheat roots.

Metabolite names	Metabolite concentrations (ng/g FW)			
	Fielder NP	Fielder LP	Ardito NP	Ardito LP
Indole-3-acetic acid (IAA)	10.69 ± 0.24b	13.39 ± 0.58a	11.54 ± 1.04b	7.39 ± 0.62c
*N*-(3-indolylacetyl)-l-alanine (IAAla)	7.33 ± 0.16a	7.27 ± 0.12a	7.18 ± 0.15a	9.56 ± 0.27b
Indole-3-acetyl-l-asparticacid (IAAsp)	0.61 ± 0.08b	0.45 ± 0.06b	0.88 ± 0.12a	0.57 ± 0.08b
*N*-(3-indolylacetyl)-l-leucine (IALeu)	0.06 ± 0.01bc	0.11 ± 0.03a	0.06 ± 0.01b	0.03 ± 0.01c
*N*-(3-indolylacetyl)-l-phenylalanine (IAPhe)	0.24 ± 0.04a	0.13 ± 0.01c	0.18 ± 0.01b	0.09 ± 0.01c
Indole-3-acetyl-l-tryptophan (IATrp)	0.06 ± 0.01a	0.06 ± 0.01a	0.07 ± 0.01a	0.04 ± 0.00b
3-Indolepropionicacid (IPA)	0.30 ± 0.04b	0.22 ± 0.02b	1.51 ± 0.20a	1.33 ± 0.24a
Total auxin	19.29 ± 0.4b	21.63 ± 0.6a	21.43 ± 1.10a	18.01 ± 0.3c
Abscisic acid (ABA)	1.14 ± 0.12a	0.69 ± 0.07b	0.71 ± 0.13b	0.42 ± 0.03c
Gibberellina1 (GA1)	0.34 ± 0.07bc	0.52 ± 0.09a	0.49 ± 0.10ab	0.284 ± 0.05c
Gibberellina3 (GA3)	0.64 ± 0.07ab	0.39 ± 0.07c	0.75 ± 0.11a	0.57 ± 0.05b
Gibberellina4 (GA4)	0.31 ± 0.03c	0.29 ± 0.07c	0.67 ± 0.03a	0.53 ± 0.04b
Gibberellina7 (GA7)	2.66 ± 0.55a	3.10 ± 0.38a	1.44 ± 0.02b	2.66 ± 0.79a
Gibberellina8 (GA8)	2.97 ± 0.58b	4.34 ± 0.16a	1.43 ± 0.03c	2.35 ± 0.54b
Gibberellina9 (GA9)	0.689 ± 0.18bc	0.473 ± 0.01c	0.76 ± 0.13b	1.41 ± 0.16a
Total GA	8.76 ± 0.64a	9.82 ± 0.28a	6.24 ± 0.42b	8.21 ± 0.51c
Uniconazole (UCZ)	0.08 ± 0.01b	0.03 ± 0.00	0.16 ± 0.04a	0.02 ± 0.01c
Paclobutrazol (PP333)	0.96 ± 0.05c	0.64 ± 0.05d	1.50 ± 0.16a	1.31 ± 0.04b
Jasmonic acid (JA)	6.39 ± 0.34c	8.62 ± 0.35a	7.23 ± 0.11b	7.17 ± 0.18b
Methyl jasmonate (MeJA)	5.14 ± 0.34b	2.89 ± 0.12c	8.32 ± 0.50a	4.44 ± 0.56b
12-oxo phytodienoic acid (OPDA)	3.04 ± 0.92b	4.94 ± 0.43a	2.78 ± 0.28b	3.61 ± 0.31b
Total JA	14.43 ± 0.44c	16.45 ± 0.48b	18.33 ± 0.82a	15.22 ± 0.54c
Salicylic acid (SA)	12.27 ± 0.41ab	13.27 ± 0.25	9.81 ± 1.13c	11.86 ± 0.67b
*meta*-TOPOLIN (mT)	0.81 ± 0.09a	0.37 ± 0.05c	0.62 ± 0.10b	0.66 ± 0.09b
*cis*-Zeatin (cZ)	4.44 ± 0.23b	5.57 ± 0.35a	5.49 ± 0.68a	3.98 ± 0.45b
*cis*-Zeatin-riboside (cZR)	344.98 ± 13.31a	260.69 ± 12.44c	307.41 ± 13.64b	266.96 ± 6.40c
*trans*-Zeatin (tZ)	0.43 ± 0.05ab	0.37 ± 0.04b	0.35 ± 0.29b	0.51 ± 0.02a
*trans*-Zeatin-riboside (tZR)	5.97 ± 0.16b	2.80 ± 0.34d	6.84 ± 0.58a	4.02 ± 0.12c
Dihydrozeatin (DZ)	0.004 ± 0.001b	0.026 ± 0.002a	0.003 ± 0.001b	0.003 ± 0.001b
*N*6-isopentenyladenosine (iPR)	5.33 ± 0.80a	3.11 ± 0.49b	2.49 ± 0.24b	3.33 ± 0.33b
*N*6-Benzyladenine (6-BA)	0.22 ± 0.03bc	0.12 ± 0.02c	2.41 ± 0.52a	0.66 ± 0.17b
*N, N*’-Diphenylurea (DPU)	0.026 ± 0.005a	0.027 ± 0.002a	0.031 ± 0.005a	0.033 ± 0.004a
Total CK	362.56 ± 14.11a	273.43 ± 12.91c	325.92 ± 14.31b	280.38 ± 5.58c
Maleic hydrazide (MH)	1.38 ± 0.21a	0.45 ± 0.07b	0.30 ± 0.06b	0.34 ± 0.04b
1-Naphthaleneacetamide (1-NAD)	ND	ND	ND	0.027
4-Chlorophenoxyacetic acid (4-CPA)	4.29 ± 0.19a	0.484 ± 0.05c	1.67 ± 0.24b	0.61 ± 0.16c
2-Methoxy-5-nitrophenol sodium salt (5-NG)	16.56 ± 1.41a	15.96 ± 0.71a	15.52 ± 0.72a	15.97 ± 1.08a

Different letters in the boxes indicate a significant difference at *p* < 0.05.

*ND* not detected.

## Discussion

### PAP and PHT regulated P assimilation and remobilization

To respond to LP stress, plants have evolved lots of strategies known as phosphate starvation responses (PSR) regulated by phosphorus-starvation-induced (PSI) genes (*PHT1*, *PAP*, *SPX*)^32^. In our study, six *PAP* genes were upregulated. They had a higher expression level in P efficient genotype Fielder under LP stress, which can support the high release of phosphorus from organic phosphorus under LP stress. Plants increase the secretion of purple acid phosphatases (PAPs) into the rhizosphere to scavenge organic phosphorus for plant use under low phosphorus stress^33^. It found that *Cm-PAP10.1* and *Cm-PAP10.2*, genes encoding purple acid phosphatases, were upregulated in melon under LP stress, the activity of PAPs secreted by plant roots under LP stress was positively correlated with the degree of P deficiency^34^. In rice, *OsPAP10c* overexpression increased acid phosphatase (APase) activity by almost fivefold in both roots and leaves under low phosphorus. Meanwhile, genes encoding phosphorus transporter proteins were screened in transcriptome data, among which four DEGs of high-affinity phosphorus transporter systems (*PHT1* and *PHO1* families) were screened; three of these were significantly upregulated in P efficient genotype but not response in P inefficient genotype Ardito treated with LP. The high-affinity transporter TaPHT1;9-4B and its transcriptional regulator TaMYB4-7D contributed to efficient Pi acquisition and plant growth under Pi-limiting conditions^35^. In *Zygophyllum xanthoxylum*, *PHO1* and *PHT1* genes were upregulated in the roots, suggesting that these phosphorus transporter protein genes may play a vital role in regulating the distribution, transport, and maintenance of dynamic homeostasis of P in the plant body during P deficiency^36^. Our results that Fielder has a higher total phosphorus content both in the stem and at the root suggests that Fielder may have activated and mobilized phosphorus by secreting more PAPs and enhanced phosphorus absorption by PHTs. SPX-domain-containing proteins (SPXs) play a crucial role in the sensing, signaling, and transport of inorganic phosphate (Pi) in eukaryotes. *AtSPX1* is a phosphate-dependent inhibitor of PHR1 in *Arabidopsis*; Os*SPX1*, Os*SPX4*, and Os*SPX6* are involved in Pi starvation signaling and acting as a negative regulator of PHR in rice^9,10,37,38^. Transgenic plants overexpressing *SPX6* exhibited decreased Pi concentrations and suppression of phosphate starvation-induced (PSI) genes^37^. In our study, four *SPX* genes were expressed in different trends under LP stress; further research is needed on the function of *SPX* in response to LP stress.

### Plant hormones signal transduction in wheat root

LP stress disrupts plant hormone synthesis and distribution, consequently impacting root growth^5^. Our study reveals significant alterations in the expression of genes involved in plant hormone signal transduction, underscoring the pivotal role of hormones in wheat root development under low phosphorus stress. Under Pi starvation, heightened auxin signaling at the root tip and lateral root primordia inhibits primary root growth while stimulating lateral root formation^31^. The growth hormone-responsive transcription factor SAUR modulates root morphology and fosters lateral root development^39,40^. Similarly, increasing cytokinin (CK) is crucial in maintaining the root-to-shoot ratio, thereby reducing CK concentrations under low phosphorus conditions^30^. Previous research indicates that the transcription factor MYB62 regulates phosphate starvation response (PSR) by modulating GA metabolism and signaling^41^, suggesting the necessity for MYB TFs and GA signaling regulation in wheat to cope with low P stress. Jasmonate (JA) and salicylic acid (SA) are additional hormones crucial for integrating environmental cues and influencing root growth under LP stress^32,42^. Our study demonstrates the upregulation of genes involved in auxin, CK, GA, JA, SA, and brassinosteroid (BR) signaling in Fielder compared to Ardito under low phosphorus stress, indicating their involvement in regulating wheat root morphology in response to low-P stress. Targeted metabolome results corroborate the transcriptome data, showing increased total IAA, ABA, GA, JA, and SA in Fielder than in Ardito under LP stress (Table 4). Under LP stress, Fielder exhibited a significant increase in total auxin, total JA, and SA contents while experiencing a decrease in total ABA and total CK contents. On the other hand, Ardito showed an increase in total GA and SA contents but a decrease in total IAA, total JA, total CK, and ABA contents under LP stress. Root morphological indicators were significantly higher in Fielder compared to Ardito, indicating Fielder’s ability to expedite root development in response to stress through upregulation of gene expression and elevation of hormone contents. Brassinosteroids (BRs) are plant hormones that promote cell elongation and division, crucial for plant growth and development^43^. Recent studies have implicated brassinosteroid signaling in regulating phosphate starvation-induced malate secretion in plants^44^. Additionally, ABA and ETH signaling were observed to be downregulated in both Fielder and Ardito under low phosphorus stress. While ABA and ETH signals may negatively regulate low phosphorus stress in roots, further investigation is required to ascertain the precise mechanism.

### Secondary metabolic synthesis regulated to LP stress

Secondary metabolites, such as phenylpropanoids, flavonoids, and anthocyanins, can scavenge reactive oxygen species, delay microbial degradation of organic acids, and enhance mobilization of rhizosphere phosphorus^45–47^. Numerous DEGs were enriched in the phenylpropanoid and flavonoid metabolism pathways, suggesting that P starvation might affect the metabolic product content of secondary metabolic synthesis in wheat roots. In our study, *PAL*, *CYP73A*, and *4CL* in the phenylpropanoid metabolism pathway were upregulated under LP stress, and *E2.3.1.133*, *PCMT*, and *CYP75A* were significantly upregulated in the comparison Fielder with Ardito, *F3H* was exclusively upregulated in Fielder and *E5.5.1.6* exclusively upregulated in Ardito suggesting the increase of genes related to flavonoid metabolism may be enhanced the low P tolerance in Fielder. Similar to our results, flavonoids were highly accumulated in P-efficient cotton genotype Jimain169 roots under low phosphorus due to the upregulation of the genes responsible for flavonoids^48^. In style, a set of genes involved in flavonoid synthesis were found to be upregulated by Pi starvation accompanied by the flavonoid metabolites, phenolic acids, and phenyl amides contents were increased in roots, which might facilitate P solubilization and cooperate with beneficial microorganisms in the rhizosphere, and thus contributing to P acquisition and utilization^49^. The above results indicate that Pi starvation altered the gene expression related to flavonoid biosynthesis. Genes and metabolites that regulate flavonoid metabolism might regulate phosphorus signaling and improve wheat tolerance to low phosphorus stress. However, the flavonoid content decreased significantly in P-resistant maize roots under low phosphorus stress, which may be attributed to the levels of phosphorus supply^50^. This may be related to the supply of phosphorus levels. In soybeans, there is more anthocyanin production in LP and more isoflavonoid production in NP^51^. Anthocyanin is also one of the most observed flavonoids in the roots and shoots under LP stress. Among various functions of flavonoids in plants, the higher accumulation of anthocyanin in leaves has a vital role in the photo-protection of the leaf. However, the function of anthocyanin in the roots is still to be elucidated^52^.

### Changes in carbohydrate biosynthesis under LP stress

Plants produce ATP and CO_2_ through photosynthetic product breakdown (e.g., glucose) by respiration to promote root growth and development or maintain root activity for nutrient and water uptake and translocation^53^. Higher root biomass and physiology are required for higher production and are important traits supporting normal plant growth under stress conditions. P‐deficiency was reported to increase carbohydrate translocation via the phloem to roots to favor root growth for better acquisition of Pi from soil^54^. Fielder has a higher root morphology than Ardito under low phosphorus stress but at the cost of an increased response to root-to-shoot ratio. In the previous study, the P efficient genotype had a more remarkable ability to maintain phosphorylated sugars (i.e., glucose-6-P and fructose-6P) by upregulating the genes involved in glycolysis, starch, and sucrose synthesis that are important for glycolysis as well as the biosynthesis of sugars and starch^55,56^. The above results suggest that wheat roots maintain ATP supply by continuously altering the transcription levels of genes encoding key enzymes in the glycolysis/gluconeogenesis pathway. Under low P conditions, *SUS*, *UGP*, *GALM*, *HK*, *PFP*, *ALDO*, and *BAM* were upregulated in Fielder, suggesting that Fielder better maintains the phosphorylated sugars required for sugar and starch biosynthesis to regulate the metabolic processes required for energy and carbon skeleton production to supply root growth^57^.

Plants secrete organic acids from TCA cycle derivatives (Major citrate and malate) to help solubilize sparingly soluble inorganic phosphate and mobilize Pi from the soil^58^. Citrate exudation from roots increased P uptake from Fe-P in soils^59^. Similarly, in the case of GhmMDH1 from cotton (*Gossypium hirsutum* L.), overexpressed cotton showed higher malate exudation and stimulation of P uptake from sparingly soluble forms^60^. It found that transgenic rice of maize PEPC showed a more significant increase of oxalate exudation and accumulated P than the wild type^61^. ATP-citrate lyase (ACLY) could catalyze the transformation among acetyl-CoA, oxaloacetate, and citrate, the key node of pyruvate metabolism going into the TCA cycle under low phosphorus. Here, *ACLY* showed a higher expression in the Fielder roots than Ardito and perhaps played a key role in promoting the TCA cycle. ACO catalyzes the transformation among isocitrate, cis-Aconitate, and citrate, also upregulated in Fielder vs. Ardito, suggesting that wheat roots mainly mobilize P absorption through the secretion of citric acid under LP stress in Fielder. However, the MDH2 was downregulated compared to Fielder with Ardito, perhaps due to energy balance.

### Transcription factors responded to LP tolerance in wheat

The transcriptional regulation of PSR involves several transcription factors (TFs), including members of the MYB, WRKY, and bHLH families. These TFs positively or negatively regulate Pi signaling and Pi homeostasis in plants^11^. In our study, the identification of 426 transcription factors suggested complex regulation in wheat root response to LP stress (Table 3). Among those identified transcription factors, 82 DEGs belonging to the MYB transcription factor family were significantly expressed by LP stress. For instance, a MYB gene (TraesCS7D02G295400) showed a significantly different expression in three comparisons (Table S9-1), indicating that MYB transcription factors are crucial in responding to low phosphorus stress. OsMYB4P- and OsMYB2P-1-overexpression upregulated the expression of the Pi transporter genes, leading to higher Pi accumulation in shoots and roots^62,63^. In addition, MYB genes have also been shown to respond to low phosphorus levels by modulating gibberellin (GA) metabolism and signaling and regulation of the miR399f promoter^41,64^.

Forty-four transcription factor genes belonging to the bHLH family were significantly expressed by LP stress. One bHLH gene (TraesCS5A02G049600) was downregulated in Fielder, Ardito, and Fielder vs. Ardito under low phosphorus stress, suggesting that wheat tends to downregulate bHLH in response to low phosphorus stress. It has also been found in other plants. For instance, AtbHLH32 is a negative regulator of several Pi-starvation responses in *Arabidopsis*. The bHLH TF OsPTF1 (Pi starvation-induced transcription factor 1) was reported to be involved in the PSR in rice. *OsPTF1*-overexpression enhanced tolerance to Pi starvation in transgenic rice by modulating root architecture rather than triggering increased expression of phosphate transporters^65^.

Additionally, 30 transcription factors belonging to the WRKY family were identified in this study. Most WRKY genes showed significant upregulation, especially in the comparison between Felder and Ardito, suggesting the WRKY may have a positive regulatory effect on low phosphorus tolerance in Fielder. WRKY45 and WRKY75 positively regulate the expression of *PHT1;1*. RNA interference (RNAi) lines of both WRKY45 and WRKY75 displayed impaired Pi uptake^66,67^. In rice, transgenic seedlings overexpressing OsWRKY74 display increased Pi uptake, longer roots, increased biomass, and higher iron accumulation levels^68^, WRKY21 and WRKY108 activate the expression of *OsPHT1;1* under Pi-sufficient conditions to promote Pi accumulation^69^. The vast and complex regulatory mechanisms indicate that TFs play an important role in responding to low phosphorus stress, and further research is needed on these TFs.

## Materials and methods

### Plant materials and treatment

Two wheat genotypes with different responses to low phosphorus (LP), including Fielder (P efficient) and Ardito (P inefficient), were used in this study. Wheat seeds were surface-sterilized with 0.5% NaClO (v/v) solution for 20 min, rinsed with sterile water, and allowed to germinate in the wet paper towel for 4–5 days at 4 °C before the seedlings were transplanted into continuously aerated 1/5 Hoagland hydroponic solution (pH 4.2) for a 2-day acclimation to low pH in a walk-in growth chamber with a stable temperature of 23 °C, a 14 h photoperiod of 400 μmol m^−2^·s^−1^ illumination level, and relative humidity of 60–80%. For different Pi-level treatments, germinated seedlings were moved into the solutions with two concentrations of NaH_2_PO_4_: 200 μmol/L (normal Pi-level, NP) or 2 μmol/L (low Pi-level, LP) and grow for 4 days. The experimental samples are entirely random and arranged with at least three replicates.

### Determination of phenotypic indicators

The plant height of seedlings was measured with a ruler, and Electronic vernier calipers were used to measure stem thickness. The relative chlorophyll content in fully developed leaves, as indicated by SPAD (Soil and Plant Analyzer Development) value, was determined with a portable Konica SPAD-502Plus instrument (Konica Minolta Holdings Inc., Tokyo, Japan). Fresh plant roots from different treatment groups were collected and completely expanded onto the scanner platform (Epson Expression 1000XL, Seiko Epson Corporation, Nagano, Japan). Analyze the scanned root photos using WinRhizo Pro software (Regent Instruments Inc., Quebec, Canada) to obtain the roots’ total length, diameter, surface area, and volume. Afterward, dry the stem and root samples at 75 ° C for 4 days and further determine the dry mass of the stem and roots using an electronic balance.

### P concentration determination

Oven-dried shoot and root samples were ground into fine powder for P concentration analysis. A 50-microgram sample was weighed out and digested with 9 mL nitric acid and 1 mL H_2_O_2_ using a microwave digestion instrument, and phosphorus concentration was measured by ICP-AES (Juguang Co., Beijing, China). Total phosphorus in the shoot (TPL) was calculated based on shoot phosphorus concentration (LPC) and shoot weight. Total phosphorus in roots (TPR) was calculated based on root phosphorus concentration (RPC) and root weight^70^.

### RNA extraction, library preparation, sequencing, and read mapping

Follow the instructions of the TRIzol kit (Invitrogen Co., Carlsbad, CA, USA) to extract total RNA from a 1 cm root tip. RNA quality and integrity were measured using a 5300 bioanalyzer (Agilent Co., Santa Clara, CA, USA) and quantified using ND-2000 (Thermo Fisher NanoDrop, Waltham, MA, USA). Only high-quality RNA samples (*OD260/280* = 1.8–2.2, *OD260/230* ≥ 2.0, > 1 μg, RIN ≥ 6.5, 28S: 18S ≥ 1.0) were used to construct sequencing library and verified by qRT-PCR. Entrust Shanghai Majorbio Biopharmaceutical Biotechnology Co., Ltd. (Shanghai, China) to perform RNA purification, reverse transcription, and library construction according to the manufacturer’s instructions (Illumina, San Diego, CA, USA), followed by sequencing analysis. 12 RNA-seq transcriptome libraries (2 genotypes × 2 treatments × 3 biological replicates) were prepared and sequenced. The FASTP^71^ soft was used to remove low-quality readings from the originally paired ends, and HISAT2^72^ (Ver. 2.2.1) was used to map clean readings onto the wheat genome for comparison. The StringTie^73^ (Ver. 1.3.6) was then used to assemble the mapped reads in a reference-based approach. All genes were annotated against public databases, including NR, Pfam, COG, Swiss-Prot, KEGG, and GO.

### Differential expression and functional enrichment analysis

Express the level of each transcript using Fragments Per Kilobases Per Millionreads (FPKM). RSEM^74^ was used to quantify gene abundances. Differential expression analysis was performed using the DESeq2^75^. DEGs with |log_2_FC| ≥ 1 and FDR ≤ 0.05 were considered significantly different expressed genes. Cluster heat diagrams were drawn using Toolkit for Biologists (TBtools Ver. 2.030) with default settings^76^. Those data were added to the Comprehensive Gene Expression Database with the accession number PRJNA1033153. In addition, Goatools and KOBAS63^77^ were used to analyze these DEGs’ GO and KEGG functions. When the Bonferroni corrected, the *p*-value was ≤ 0.05, and GO terms, and KEGG metabolic pathways were significantly enriched compared to the entire transcriptome background.

### Metabolome detection

The frozen wheat root apices were dispatched to Shanghai Majorbio Biopharmaceutical Biotechnology Co., Ltd. (Shanghai, China) for phytohormone extraction and analysis. Precision weighing was conducted on 100 mg root samples, which were then placed into 2 mL grinding tubes. Subsequently, 498 μL of 80% methanol and 2 μL of SA-D4 internal standard solution (2 μg/mL) were added to each tube. The mixture was then ground at low temperature for 3 minutes and subjected to ultrasound extraction at low temperature for 1 hour. To the extracted sample, 25 mg of EN15662 (consisting of magnesium sulfate 4 g, sodium chloride 1 g, sodium citrate 2 hydrate 1 g, disodium hydrogen citrate 1.5 hydrate 0.5 g) was added as a salt packet. The mixture was immediately shaken and vigorously vibrated on an oscillator for 10 minutes. Following this, it was centrifuged at 10 °C for 10 min, and 100 μL of supernatant was extracted. To this, 60 μL of water was added, and the solution was thoroughly swirled before transferring it into a sample vial. Qualitative and quantitative detection of the target substances in the samples was carried out using LC-ESI-MS/MS (UHPLC-Qtrap). The specific parameters included a WatersBEHC18 (2.1 × 100 mm, 1.7 μm) liquid chromatography column with a column temperature of 30 °C and a sample size of 10 μL. The mobile phase consisted of mobile phase A (0.1% formic acid water) and mobile phase B (0.1% formic acid acetonitrile), with a balance time of 3 min and a collection time of 10 min. In Sciex quantitative software OS, default parameters were utilized to automatically identify and integrate each ion fragment and assist in manual inspection. A standard linear regression curve was established with the mass peak area of the analyte as the ordinate and the analyte concentration as the abscissa. For sample concentration calculation, the mass spectrum peak area of the sample analyte was substituted into the linear equation to determine the concentration result.

### Quantitative real-time PCR (qRT-PCR) validation

Using the identical RNA/cDNAs for RNA-seq as templates, qRT-PCR was performed on a Bio-Rad CFX96 (Bio-Rad Laboratories, Hercules, CA, USA) to verify the authenticity of transcriptomic profile expression patterns. The 10 μL reaction system contained TB Green^®^ Premix Ex Taq™ II (Takara standard Co., Osaka, Japan) 5 μL, 10 μM primers each 0.2 μL, cDNA template 1 μL, and ddH_2_O 3.6 μL. The amplification procedure was initially 95 °C for 30 s, 40 cycles of 95 °C for 10 s, and 60 °C for 30 s (two-step thermal cycling). Twenty candidate DEGs involved in various processes were randomly picked up as target genes, and the housekeeping gene *TaActin* was used as an internal control. The primers used for qRT-PCR are listed in Table S1. The expression level of the gene was determined using the 2^−ΔΔCT^ formula^78^.

### Statistical analysis

Analysis of variance (ANOVA) followed by Duncan’s multiple range test (DMRT) was conducted to compare means among treatments using SPSS software (version 19.0). Before ANOVA, the data underwent normality testing using Chi-square analysis^79^.

### Ethics declarations

The authors declare no competing interests. This study does not include human or animal subjects. All experimental research and studies on plants (wheat cultivars) comply with relevant institutional, national, and international guidelines and legislation, as well as the IUCN Policy Statement on Research Involving Species at Risk of Extinction and the Convention on the Trade in Endangered Species of Wild Fauna and Flora.

### Supplementary Information

Supplementary Figures.

Supplementary Table S1.

Supplementary Table S2.

Supplementary Table S3.

Supplementary Table S4.

Supplementary Table S5.

Supplementary Table S6.

Supplementary Table S7.

Supplementary Table S8.

Supplementary Table S9.

## Data Availability

The original contributions presented in the study are available in the article or Supplementary. The RAN-seq raw data can be found on the NCBI repository, accession number PRJNA1033153. Supplementary material associated with this article can be found in the online version.
