# Supplementary material for: Comparative transcriptomic and physiological analyses unravel wheat source root adaptation to phosphorous deficiency
Source: Sci Rep. 2024 May 14;14:11050. doi: 10.1038/s41598-024-61767-z (PMC11094128; doi:10.1038/s41598-024-61767-z)
Supplement: Supplementary file 1 — Supplementary Figures. [file 41598_2024_61767_MOESM1_ESM.pdf]

Comparative transcriptomic and physiological analyses unravel wheat source root adaptation to phosphorous deficiency

Daozhen Luo [1], Muhammad Usman [1], Fei, Pang [1], Wenjie Zhang [1], Ying Qin [1], Qing Li [1], Yangrui Li [2], Yongxiu Xing [1], and Dengfeng Dong [1,*]

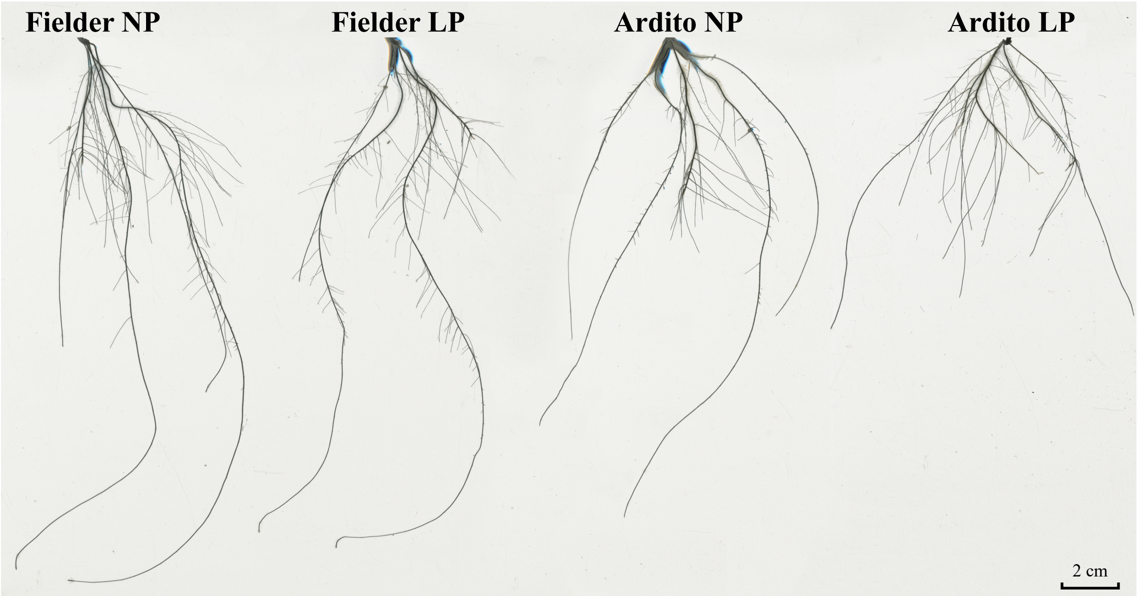

Fig. S1. Representative pictures of root morphology of two different wheat varieties (Fielder and Ardito) under normal and low phosphorous conditions. NP show normal phosphorous and LP indicates low phosphorous.

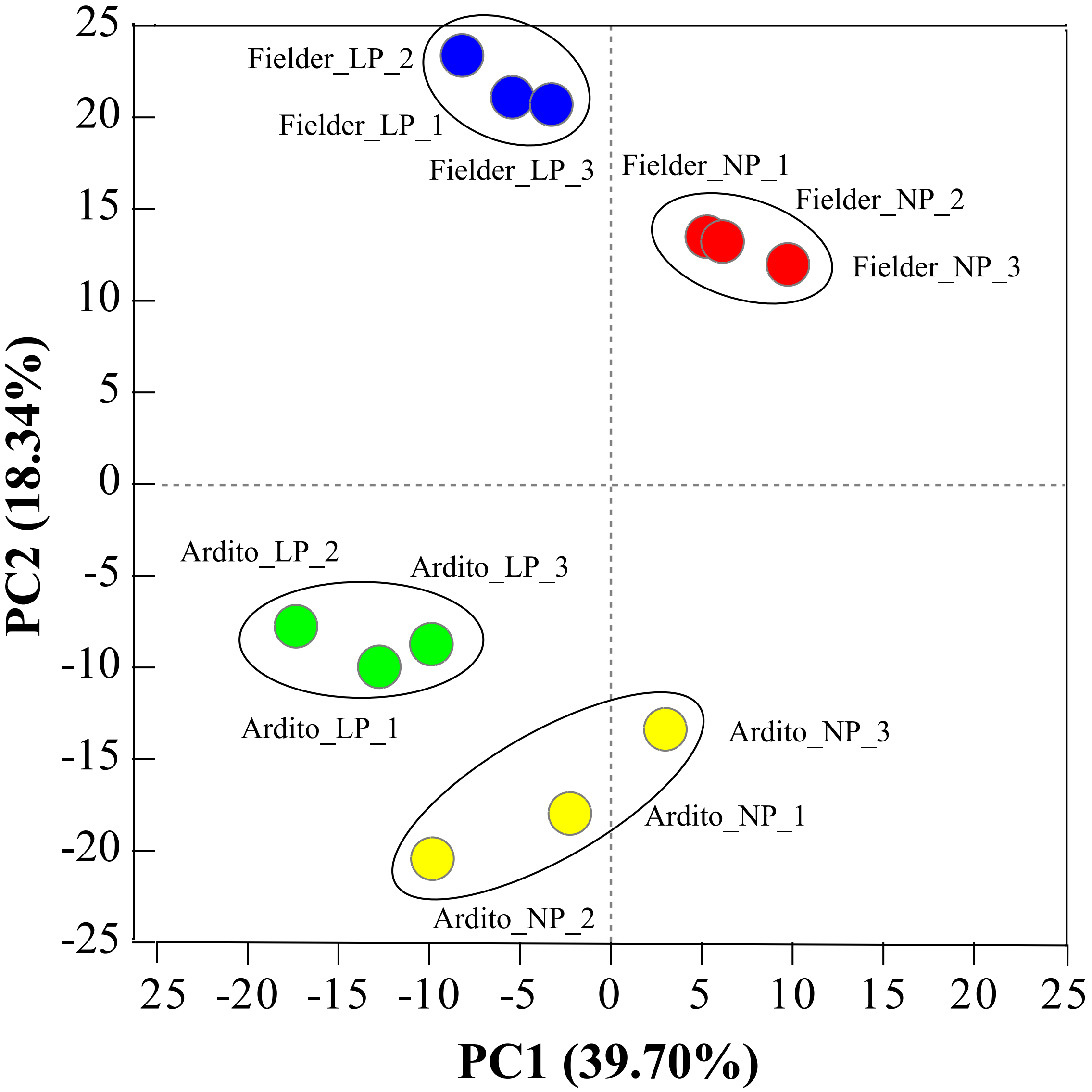

Fig. S2. Principle component analysis plot of transcriptome profiles under different phosphorous conditions. NP indicates normal phosphorous and LP indicates low phosphorous.
